# Supplementary material for: Belief in sexism shift: Defining a new form of contemporary sexism and introducing the belief in sexism shift scale (BSS scale)
Source: PLoS One. 2021 Mar 11;16(3):e0248374. doi: 10.1371/journal.pone.0248374 (PMC7951888; doi:10.1371/journal.pone.0248374)
Supplement: S1 Table — (DOCX) [file pone.0248374.s001.docx]

**Belief in Sexism Shift:**

**Defining a new form of contemporary sexism and introducing the belief in sexism shift scale (BSS scale)**

Supporting information 1

Miriam K. Zehnter, Francesca Manzi, Patrick E. Shrout, and Madeline E. Heilman

| **S1 Table. Standardized component and factor loadings of all items by study** | | | | |
| --- | --- | --- | --- | --- |
|  | **Pilot**  (N = 112) | **Study 1**  (N = 220) | | **Study 2**  (N = 335) |
|  | PCA | EFA | CFA | CFA |
| 1. In the US, discrimination against men is on the rise. | .90 | .86 | .86 | .87 |
| 1. Men are not particularly discriminated against.* | -.81 | -.54 | -.55 | -.68 |
| 1. If anything, men are more discriminated against than women these days. | .88 | .87 | .86 | .91 |
| 1. Giving women more rights often requires taking away men’s rights. | .86 | .79 | .79 | 75 |
| 1. Under the guise of equality for women, men are actually being discriminated against. | .88 | .91 | .91 | .92 |
| 1. In the pursuit of women’s rights, the government has neglected men’s rights. | .91 | .88 | .89 | .87 |
| 1. Nowadays, men don’t have the same chances in the job market as women. | .80 | .79 | .78 | .77 |
| 1. Feminism is about favoring women over men. | .80 | .79 | .78 | .83 |
| 1. Feminism does not discriminate against men.* | -.76 | -.66 | -.66 | -.74 |
| 1. All in all, men have more responsibilities and fewer benefits. | .84 | .81 | .81 | .83 |
| 1. In today’s society, women can say things that men are not allowed to say. | .78 | .75 | .75 | .68 |
| 1. It is evident that the media is biased against men. | .92 | .83 | .84 | .87 |
| 1. In today’s society, men are often punished for acting manly. | .80 | .82 | .82 | .83 |
| 1. All in all, men are well respected in today’s society.* | -.69 | -.58 | -.59 | -.61 |
| 1. While women can use the “gender-card” to get ahead, men can’t. | .73 | .79 | .79 | .79 |
| 1. Giving more rights to women does not result in taking men’s rights away.* | -.76 | -.65 |  |  |
| 1. Nowadays, men often miss out on good jobs due to affirmative action for women. | .82 | .80 |  |  |
| 1. Due to feminism, women get more than they deserve. | .86 | .90 |  |  |
| 1. Investing money in maternity leave and childcare support means that men receive less. | .80 | .68 |  |  |
| 1. Men are demanded to be more politically correct than women. | .74 | .76 |  |  |
| 1. It is unfair to men when women interpret innocent remarks as sexist. | .69 | .67 |  |  |
| 1. In general, the media treats women more leniently than it treats men. | .85 | .76 |  |  |
| 1. It’s rare to see men treated in a sexist manner on television.* | -.61 | -.33 |  |  |
| 1. Today’s society values femininity more than masculinity. | .76 | .82 |  |  |
| 1. While women can use the “victim- card” to get ahead, men can’t. | .73 | .78 |  |  |
| 1. Women have gained too much power over men’s rights. | .89 | .88 |  |  |
| 1. Women have much more power over men than people generally assume. | .86 | .82 |  |  |
| 1. Women have little power over men’s rights.* | -.64 | -.42 |  |  |
| 1. Today is not a good time to be a man. | .69 |  |  |  |
| 1. Discrimination against men has become quite strong in the US. | .91 |  |  |  |
| 1. It’s easy to understand the anger of men’s groups in the US. | .87 |  |  |  |
| 1. Discrimination against men is not a real issue in the US.* | -.66 |  |  |  |
| 1. Today, men actually suffer more discrimination than women. | .86 |  |  |  |
| 1. Generally, women enjoy more privileges than men. | .80 |  |  |  |
| 1. Nowadays, it is easier to be a woman than a man. | .81 |  |  |  |
| 1. Men don’t suffer more discrimination than women.* | -.64 |  |  |  |
| 1. Generally, it is easier to be a man than a woman.* | -.66 |  |  |  |
| 1. All in all, men are not more disadvantaged than women.* | -.46 |  |  |  |
| 1. As discrimination against women has become weaker, discrimination against men has become stronger. | .89 |  |  |  |
| 1. The government should focus more on men’s rights. | .76 |  |  |  |
| 1. In trying to achieve equality for women, the government has treated men unfairly. | .88 |  |  |  |
| 1. Over the past few years, the government has shown too little concern about the treatment of men. | .88 |  |  |  |
| 1. Women now have more opportunities than men. | .81 |  |  |  |
| 1. To increase the number of women, less qualified women are favored over more qualified men for many jobs. | .77 |  |  |  |
| 1. Many women in the workforce are taking jobs away from men who need them more. | .77 |  |  |  |
| 1. Unemployment rates among men are rising, because so many women now enter the workforce. | .72 |  |  |  |
| 1. Today, there are no jobs left for real men. | .60 |  |  |  |
| 1. Generally, women are not favored over men in today’s job market.* | -.56 |  |  |  |
| 1. Qualified men can find good jobs regardless of affirmative action for women.* | -.57 |  |  |  |
| 1. Feminism has helped women too much. | .74 |  |  |  |
| 1. Feminism wants to take away men’s right. | .86 |  |  |  |
| 1. Cuts in social services affect men more than women. | .50 |  |  |  |
| 1. Too many social resources are going into providing childcare for women. | .74 |  |  |  |
| 1. Men are often required to pay too much childcare support. | .60 |  |  |  |
| 1. Women are too easily offended when men don’t express themselves in a politically correct manner. | .75 |  |  |  |
| 1. Women generally understand that sexist comments can be a joke. | -.09 |  |  |  |
| 1. Overall, political correctness does not limit men’s freedom of speech.* | -.74 |  |  |  |
| 1. The media should be more concerned about men’s rights. | .84 |  |  |  |
| 1. The media does not take female violence against men seriously enough. | .50 |  |  |  |
| 1. In the media, men are often the bad guys and women the good ones. | .76 |  |  |  |
| 1. In the US, the media often portrays men more negatively than it portrays women. | .82 |  |  |  |
| 1. Most media outlets are not biased against men.* | -.65 |  |  |  |
| 1. Masculinity is still highly valued in today’s society. | -.65 |  |  |  |
| 1. Nowadays, women actually have more influence in American politics than men. | .68 |  |  |  |
| 1. Women do not have more influence in American politics than men.* | -.56 |  |  |  |
| 1. Liberals want to take more and more of men’s rights away. | .87 |  |  |  |
| 1. If liberals really care about equality, they should focus more on men’s rights. | .89 |  |  |  |
| 1. While liberals pursue women’s rights, they take men’s rights away. | .90 |  |  |  |
| 1. Liberals want to take rights away from men to give them to women. | .86 |  |  |  |
| 1. Liberal policies do not discriminate against men.* | -.78 |  |  |  |
| 1. While men have to work hard, women just have to look pretty to get ahead. | .62 |  |  |  |
| 1. It is unfair to men that women can use their female charms to get ahead. | .70 |  |  |  |
| 1. While women can use sex to get ahead, men can’t. | .68 |  |  |  |
| 1. Men are often powerless against sexual harassment claims made by women. | .68 |  |  |  |
| 1. It is rare that an attractive woman gets a job solely based on her looks.* | -.43 |  |  |  |
| Note. * signifies reverse-coded items | | | | |
