# Supplementary material for: Belief in sexism shift: Defining a new form of contemporary sexism and introducing the belief in sexism shift scale (BSS scale)
Source: PLoS One. 2021 Mar 11;16(3):e0248374. doi: 10.1371/journal.pone.0248374 (PMC7951888; doi:10.1371/journal.pone.0248374)
Supplement: S2 Table. A. Study material for the pilot study. B. Study material for Study 1. C. Study material for Study 2 — (DOCX) [file pone.0248374.s002.docx]

Belief in Sexism Shift:

Defining a new form of contemporary sexism and introducing the belief in sexism shift scale (BSS scale)

Supporting information 2

Miriam K. Zehnter, Francesca Manzi, Patrick E. Shrout, and Madeline E. Heilman

| **S2 Table A. Study material for the pilot study** |
| --- |
| **Belief in sexism shift – initial item pool** |
| How much do you agree with the following statements?  There are no right or wrong answers. We are interested in *your* opinion!  1 = *I strongly disagree,* 7 = *I strongly agree* |
| 1. In the US, discrimination against men is on the rise. |
| 1. Men are not particularly discriminated against.* |
| 1. If anything, men are more discriminated against than women these days. |
| 1. Giving women more rights often requires taking away men’s rights. |
| 1. Under the guise of equality for women, men are actually being discriminated against. |
| 1. In the pursuit of women’s rights, the government has neglected men’s rights. |
| 1. Nowadays, men don’t have the same chances in the job market as women. |
| 1. Feminism is about favoring women over men. |
| 1. Feminism does not discriminate against men.* |
| 1. All in all, men have more responsibilities and fewer benefits. |
| 1. In today’s society, women can say things that men are not allowed to say. |
| 1. It is evident that the media is biased against men. |
| 1. In today’s society, men are often punished for acting manly. |
| 1. All in all, men are well respected in today’s society.* |
| 1. While women can use the “gender-card” to get ahead, men can’t. |
| 1. Giving more rights to women does not result in taking men’s rights away.* |
| 1. Nowadays, men often miss out on good jobs due to affirmative action for women. |
| 1. Due to feminism, women get more than they deserve. |
| 1. Investing money in maternity leave and childcare support means that men receive less. |
| 1. Men are demanded to be more politically correct than women. |
| 1. It is unfair to men when women interpret innocent remarks as sexist. |
| 1. In general, the media treats women more leniently than it treats men. |
| 1. It’s rare to see men treated in a sexist manner on television.* |
| 1. Today’s society values femininity more than masculinity. |
| 1. While women can use the “victim- card” to get ahead, men can’t. |
| 1. Women have gained too much power over men’s rights. |
| 1. Women have much more power over men than people generally assume. |
| 1. Women have little power over men’s rights.* |
| 1. Today is not a good time to be a man. |
| 1. Discrimination against men has become quite strong in the US. |
| 1. It’s easy to understand the anger of men’s groups in the US. |
| 1. Discrimination against men is not a real issue in the US.* |
| 1. Today, men actually suffer more discrimination than women. |
| 1. Generally, women enjoy more privileges than men. |
| 1. Nowadays, it is easier to be a woman than a man. |
| 1. Men don’t suffer more discrimination than women.* |
| 1. Generally, it is easier to be a man than a woman.* |
| 1. All in all, men are not more disadvantaged than women.* |
| 1. As discrimination against women has become weaker, discrimination against men has become stronger. |
| 1. The government should focus more on men’s rights. |
| 1. In trying to achieve equality for women, the government has treated men unfairly. |
| 1. Over the past few years, the government has shown too little concern about the treatment of men. |
| 1. Women now have more opportunities than men. |
| 1. To increase the number of women, less qualified women are favored over more qualified men for many jobs. |
| 1. Many women in the workforce are taking jobs away from men who need them more. |
| 1. Unemployment rates among men are rising, because so many women now enter the workforce. |
| 1. Today, there are no jobs left for real men. |
| 1. Generally, women are not favored over men in today’s job market.* |
| 1. Qualified men can find good jobs regardless of affirmative action for women.* |
| 1. Feminism has helped women too much. |
| 1. Feminism wants to take away men’s right. |
| 1. Cuts in social services affect men more than women. |
| 1. Too many social resources are going into providing childcare for women. |
| 1. Men are often required to pay too much childcare support. |
| 1. Women are too easily offended when men don’t express themselves in a politically correct manner. |
| 1. Women generally understand that sexist comments can be a joke. |
| 1. Overall, political correctness does not limit men’s freedom of speech.* |
| 1. The media should be more concerned about men’s rights. |
| 1. The media does not take female violence against men seriously enough. |
| 1. In the media, men are often the bad guys and women the good ones. |
| 1. In the US, the media often portrays men more negatively than it portrays women. |
| 1. Most media outlets are not biased against men.* |
| 1. Masculinity is still highly valued in today’s society. |
| 1. Nowadays, women actually have more influence in American politics than men. |
| 1. Women do not have more influence in American politics than men.* |
| 1. Liberals want to take more and more of men’s rights away. |
| 1. If liberals really care about equality, they should focus more on men’s rights. |
| 1. While liberals pursue women’s rights, they take men’s rights away. |
| 1. Liberals want to take rights away from men to give them to women. |
| 1. Liberal policies do not discriminate against men.* |
| 1. While men have to work hard, women just have to look pretty to get ahead. |
| 1. It is unfair to men that women can use their female charms to get ahead. |
| 1. While women can use sex to get ahead, men can’t. |
| 1. Men are often powerless against sexual harassment claims made by women. |
| 1. It is rare that an attractive woman gets a job solely based on her looks.* |
| **Instructed response items** |
| 1. Reading the items carefully is critical, if you are paying attention please choose "I strongly agree". |
| 1. To show that you are still paying attention, please select "I strongly disagree". |
| Note. * signifies reverse-coded items |

| **S2 Table B. Study material for Study 1** |
| --- |
| **Belief in sexism shift – reduced item pool** |
| How much do you agree with the following statements?  There are no right or wrong answers. We are interested in *your* opinion!  1 = *I strongly disagree,* 7 = *I strongly agree* |
| 1. In the US, discrimination against men is on the rise. |
| 1. Men are not particularly discriminated against.* |
| 1. If anything, men are more discriminated against than women these days. |
| 1. Giving women more rights often requires taking away men’s rights. |
| 1. Under the guise of equality for women, men are actually being discriminated against. |
| 1. In the pursuit of women’s rights, the government has neglected men’s rights. |
| 1. Nowadays, men don’t have the same chances in the job market as women. |
| 1. Feminism is about favoring women over men. |
| 1. Feminism does not discriminate against men.* |
| 1. All in all, men have more responsibilities and fewer benefits. |
| 1. In today’s society, women can say things that men are not allowed to say. |
| 1. It is evident that the media is biased against men. |
| 1. In today’s society, men are often punished for acting manly. |
| 1. All in all, men are well respected in today’s society.* |
| 1. While women can use the “gender-card” to get ahead, men can’t. |
| 1. Giving more rights to women does not result in taking men’s rights away.* |
| 1. Nowadays, men often miss out on good jobs due to affirmative action for women. |
| 1. Due to feminism, women get more than they deserve. |
| 1. Investing money in maternity leave and childcare support means that men receive less. |
| 1. Men are demanded to be more politically correct than women. |
| 1. It is unfair to men when women interpret innocent remarks as sexist. |
| 1. In general, the media treats women more leniently than it treats men. |
| 1. It’s rare to see men treated in a sexist manner on television.* |
| 1. Today’s society values femininity more than masculinity. |
| 1. While women can use the “victim- card” to get ahead, men can’t. |
| 1. Women have gained too much power over men’s rights. |
| 1. Women have much more power over men than people generally assume. |
| 1. Women have little power over men’s rights.* |
| **Instructed response item** |
| 1. Reading the items carefully is critical, if you are paying attention please choose "I strongly agree". |
| Note. * signifies reverse-coded items |

| **S2 Table C. Study material for Study 2** |
| --- |
| **Belief in sexism shift scale** |
| How much do you agree with the following statements?  There are no right or wrong answers. We are interested in *your* opinion!  1 = *I strongly disagree,* 7 = *I strongly agree* |
| 1. In the US, discrimination against men is on the rise. |
| 1. Men are not particularly discriminated against.* |
| 1. If anything, men are more discriminated against than women these days. |
| 1. Giving women more rights often requires taking away men’s rights. |
| 1. Under the guise of equality for women, men are actually being discriminated against. |
| 1. In the pursuit of women’s rights, the government has neglected men’s rights. |
| 1. Nowadays, men don’t have the same chances in the job market as women. |
| 1. Feminism is about favoring women over men. |
| 1. Feminism does not discriminate against men.* |
| 1. All in all, men have more responsibilities and fewer benefits. |
| 1. In today’s society, women can say things that men are not allowed to say. |
| 1. It is evident that the media is biased against men. |
| 1. In today’s society, men are often punished for acting manly. |
| 1. All in all, men are well respected in today’s society.* |
| 1. While women can use the “gender-card” to get ahead, men can’t. |
| **Instructed response item** |
| 1. Reading the items carefully is critical, if you are paying attention please choose "I strongly agree". |
| **Attitudes towards women scale [27]** |
| How much do you agree with the following statements?  There are no right or wrong answers. We are interested in *your* opinion!  1 = *I strongly disagree,* 7 = *I strongly agree* |
| 1. Swearing and obscenity are more repulsive in the speech of a woman than of a man. |
| 1. Women should take increasing responsibility for leadership in solving the intellectual and social problems of the day.* |
| 1. Both husband and wife should be allowed the same grounds for divorce.* |
| 1. Telling dirty jokes should be mostly a masculine prerogative. |
| 1. Intoxication among women is worse than intoxication among men. |
| 1. Under modern economic conditions with women being active outside the home, men should share in household tasks such as washing dishes and doing the laundry.* |
| 1. It is insulting to women to have the "obey" clause remain in the marriage service.* |
| 1. There should be a strict merit system in job appointment and promotion without regard to sex.* |
| 1. A woman should be as free as a man to propose marriage.* |
| 1. Women should worry less about their rights and more about becoming good wives and mothers. |
| 1. Women earning as much as their dates should bear equally the expense when they go out together.* |
| 1. Women should assume their rightful place in business and all the professions along with men.* |
| 1. A woman should not expect to go to exactly the same places or to have quite the same freedom of action as a man. |
| 1. Sons in a family should be given more encouragement to go to college than daughters. |
| 1. It is ridiculous for a woman to run a locomotive and for a man to darn socks. |
| 1. In general, the father should have greater authority than the mother in the bringing up of children. |
| 1. Women should be encouraged not to become sexually intimate with anyone before marriage, even their fiancés. |
| 1. The husband should not be favored by law over the wife in the disposal of family property or income.* |
| 1. Women should be concerned with their duties of childbearing and house tending, rather than with desires for professional and business careers. |
| 1. The intellectual leadership of a community should be largely in the hands of men. |
| 1. Economic and social freedom is worth far more to women than acceptance of the ideal of femininity which has been set up by men.* |
| 1. On the average, women should be regarded as less capable of contributing to economic production than are men. |
| 1. There are many jobs in which men should be given preference over women in being hired or promoted. |
| 1. Women should be given equal opportunity with men for apprenticeship in the various trades.* |
| 1. The modern girl is entitled to the same freedom from regulation and control that is given to the modern boy.* |
| **Modern sexism scale [28]** |
| How much do you agree with the following statements?  There are no right or wrong answers. We are interested in *your* opinion!  1 = *I strongly disagree,* 7 = *I strongly agree* |
| 1. Discrimination against women is no longer a problem in the United States. |
| 1. Women often miss out on good jobs due to sexual discrimination.* |
| 1. It is rare to see women treated in a sexist manner on television. |
| 1. On average, people in our society treat husbands and wives equally. |
| 1. Society has reached the point where women and men have equal opportunities for achievement. |
| 1. It is easy to understand the anger of women's groups in America.* |
| 1. It is easy to understand why women's groups are still concerned about societal limitations of women's opportunities.* |
| 1. Over the past few years, the government and news media have been showing more concern about the treatment of women than is warranted by women's actual experiences. |
| **Ambivalent sexism inventory [29]** |
| How much do you agree with the following statements?  There are no right or wrong answers. We are interested in *your* opinion!  1 = *I strongly disagree,* 7 = *I strongly agree* |
| 1. No matter how accomplished he is, a man is not truly complete as a person unless he has the love of a woman. |
| 1. Many women are actually seeking special favors, such as hiring policies that favor them over men, under the guise of asking for "equality". |
| 1. In a disaster, women ought not necessarily to be rescued before men.* |
| 1. Most women interpret innocent remarks or acts as being sexist. |
| 1. Women are too easily offended. |
| 1. People are often truly happy in life without being romantically involved with a member of the other sex.* |
| 1. Feminists are not seeking for women to have more power than men.* |
| 1. Many women have a quality of purity that few men possess. |
| 1. Women should be cherished and protected by men. |
| 1. Most women fail to appreciate fully all that men do for them. |
| 1. Women seek to gain power by getting control over men. |
| 1. Every man ought to have a woman whom he adores. |
| 1. Men are complete without women.* |
| 1. Women exaggerate problems they have at work. |
| 1. Once a woman gets a man to commit to her, she usually tries to put him on a tight leash. |
| 1. When women lose to men in a fair competition, they typically complain about being discriminated against. |
| 1. A good woman should be set on a pedestal by her man. |
| 1. There are actually very few women who get a kick out of teasing men by seeming sexually available and then refusing male advances.* |
| 1. Women, compared to men, tend to have a superior moral sensibility. |
| 1. Men should be willing to sacrifice their own well-being in order to provide financially for the women in their lives. |
| 1. Feminists are making entirely reasonable demands of men.* |
| 1. Women, as compared to men, tend to have a more refined sense of culture and good taste. |
| **Social desirability [44]** |
| Finally, please answer the following questions about yourself.  TRUE or FALSE? |
| 1. I have never intensely disliked anyone. |
| 1. I sometimes feel resentful when I don’t get my way.* |
| 1. No matter who I am talking to, I am always a good listener. |
| 1. There have been occasions when I took advantage of someone.* |
| 1. I am always willing to admit it when I make a mistake. |
| 1. I sometimes try to get even, rather than forgive and forget.* |
| 1. There have been occasions when I felt like smashing things.* |
| 1. There have been times when I was quite jealous of the good fortune of others.* |
| 1. I have never felt that I was punished without cause. |
| 1. I have never deliberately said something that hurt someone’s feelings. |
| Note. * signifies reverse-coded items |
